# SGLT2 inhibitors for patients with heart failure with preserved ejection fraction in China: a cost-effectiveness study

**DOI:** 10.3389/fphar.2023.1155210

**Published:** 2023-09-13

**Authors:** He Lu, Pingping Shang, Dexing Zhou

**Affiliations:** The People’s Hospital of Jiawang District of Xuzhou, Xuzhou, China

**Keywords:** empagliflozin, dapagliflozin, heart failure with preserved ejection fraction, cost-effectiveness, China

## Abstract

**Background:** The potential benefits of intervention with empagliflozin or dapagliflozin for patients with heart failure with preserved ejection fraction (HFpEF) were first demonstrated in the EMPEROR-Preserved and DELIVER studies. However, the cost-effectiveness of this intervention (empagliflozin or dapagliflozin) is yet to be established.

**Methods:** In the context of Chinese healthcare, a Markov model was proposed, which incorporates clinical outcomes from the EMPEROR-Preserved and DELIVER studies, to predict the utility and costs over a lifetime. The time horizon was 20 years, and a 5% discount rate was applied to the costs and utilities. The incremental cost-effectiveness ratio (ICER) threshold against willingness to pay (WTP) was set as the primary outcome. The robustness of the decision was evaluated using sensitivity analyses.

**Results:** After a simulated 20-year lifetime, a 72-year-old patient with HFpEF in the intervention group (empagliflozin) showed an increase of 0.44 quality-adjusted life years (QALYs) and $1,623.58 with an ICER of $3,691.56 per QALY, which was lower than the WTP threshold of $12,032.10 per QALY. A 72-year-old patient with HFpEF in the intervention group (dapagliflozin) showed an increase of 0.34 QALYs and $2,002.13 with an ICER of $5,907.79 per QALY, which was lower than the WTP threshold of $12,032.10 per QALY. One-way sensitivity analyses showed that cardiovascular (CV) mortality in the intervention and comparator groups was the most sensitive to the decision. Cost-effectiveness was demonstrated in the intervention group (empagliflozin or dapagliflozin) in 67.9% or 62.2% of 1000 Monte Carlo simulations, respectively.

**Conclusion:** In Chinese healthcare, the interventions (empagliflozin or dapagliflozin) for HFpEF were more cost-effective than the comparators. Our study has provided a quantitative evaluation of the costs and benefits of such interventions for a lifetime using the model.

## Introduction

Heart failure (HF) is a great public health challenge that poses an immense global economic and social burden in rapidly aging and growing populations (Di Tanna et al., 2019). There are approximately 38 million patients with HF with substantial morbidity and mortality worldwide (Wang et al., 2021), and almost 50% of HF cases are complicated by preserved ejection fraction (Zhang et al., 2017). According to the last nationwide population study, there are approximately 12 million patients with HF in China, among whom approximately 36% are those with heart failure with preserved ejection fraction (HFpEF) (Wang et al., 2021). Although patients with HFpEF have a similar cardiovascular (CV) risk and decrease in quality of life as those with heart failure with reduced ejection fraction (HFrEF), the former is accompanied by high comorbidities including hypertension, atrial fibrillation and coronary heart disease, a high risk of hospitalisation for HF (HHF) and CV death from HFpEF, which are expected to impose an important challenge on the healthcare system in the coming decades, especially as the costs of HF treatment and management continue to rise (Mamas et al., 2017).

Evidence-based medicine has found that sodium, glucose cotransporter 2 inhibitor (SGLT2i), sacubitril/valsartan (SAC/VAL), ivabradine, vericiguat and omecamtiv may show considerable benefits for HFrEF (Lim et al., 2022), but HFpEF remains an important unmet field and effective treatment for patients remains unsolved. Empagliflozin and dapagliflozin have become the preferred drugs that have provided potential benefits to patients with HFpEF in recent years (Anker et al., 2021; Solomon et al., 2022). The EMPEROR-Preserved study (The Empagliflozin Outcome Trial in Patients with Chronic Heart Failure with Preserved Ejection Fraction) and the DELIVER study (Dapagliflozin Evaluation to Improve the Lives of Patients with Preserved Ejection Fraction Heart Failure) demonstrated a correlation between reduction in CV mortality and/or HHF events in patients with HFpEF, regardless of their diabetes status. Patients who received empagliflozin exhibited a 21% reduction in CV mortality or HHF, and the addition of dapagliflozin to standard therapy was associated with an 18% reduction in the primary composite endpoint of CV death or HHF, thus providing novel insight into HF guidelines and clinical practise (Anker et al., 2021; Solomon et al., 2022).

Understanding the cost-effectiveness of the new intervention will be practical for healthcare systems and payers, given the clinical efficacy and additional cost. To date, more research has focused on cost-effectiveness analyses of HFrEF or HF as a homogeneous group. Pharmacoeconomic research that evaluates pharmacological treatment for HFpEF is still unsolved. HFpEF populations with less severe conditions than HFrEF populations tend to receive fewer benefits when receiving expensive pharmacological therapy, thereby resulting in a higher incremental cost-effectiveness ratio (ICER). Therefore, our study conducted an independent cost-effectiveness analysis of empagliflozin and dapagliflozin in a cohort of simulated patients with HFpEF (EF ≥ 40%) based on the EMPEROR-Preserved and DELIVER studies to bridge a previous research gap.

## Methods

### Structure of the model

**FIGURE 1:**
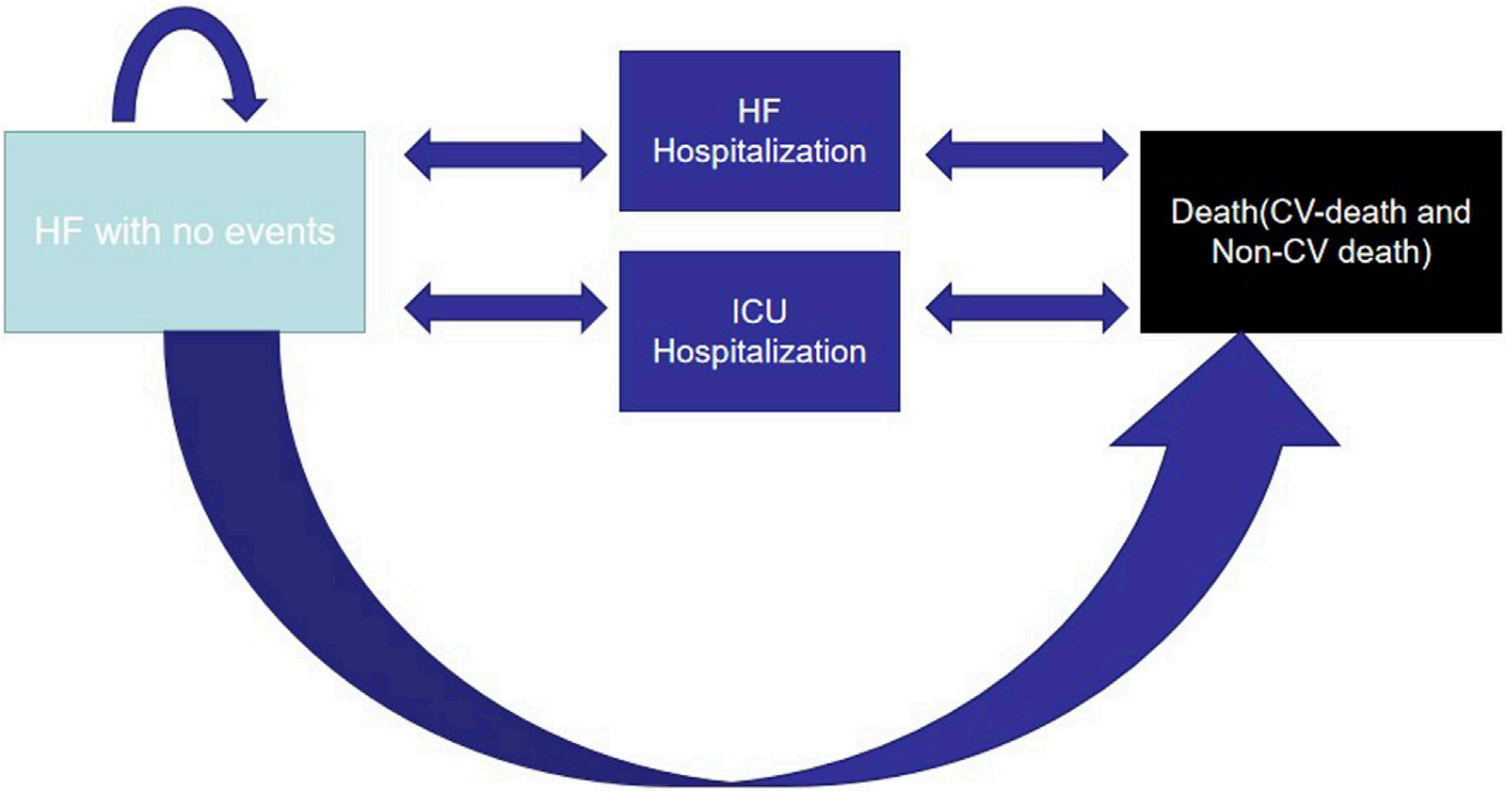
Markov model structure.

Microsoft Excel 2010 established a two-health-state Markov model to simulate the progress and prognosis of the disease, including HFpEF without events and death (CV death in the hospital, CV death and death from non-CV diseases), to compare the cost-effectiveness of interventions (empagliflozin or dapagliflozin) (Figure 1). During the 3-month cycle, owing to the characteristics of HF development, HFpEF without events was likely to progress to HHF or require an urgent visit to HF. To avoid overestimating the expected lifespan, a half-cycle correction was implemented. Costs and utilities were discounted at 5.0% annually according to “The Guidelines for Pharmacoeconomic Evaluations of China (2020)” (Liu, 2020), and a range of 0%–8% was applied in the one-way sensitivity analysis. Furthermore, it was proposed that patients with HFpEF without events entered the model at 72 years based on the average age of 103,538 Chinese patients with HFpEF and walked through the model until the simulated populations were 92 years old or died (Cai et al., 2022). The simulated population in the model received the same standard therapy, the efficacy of empagliflozin or dapagliflozin remained unchanged in 20 years, and some patients who did not tolerate the drugs stopped the therapy and caused no additional costs or utilities.

### Simulated population

The simulated population comprised patients with clinical characteristics similar to those in the EMPEROR-Preserved and DELIVER studies, including a left ventricular ejection fraction of ≥40%, HF class II–IV of the New York Heart Association and N-terminal pro-brain natriuretic peptide of >600 pg/mL. The intervention group received empagliflozin or dapagliflozin 10 mg daily as an add-on to the standard therapy for HF. The comparator group received a placebo and standard treatment for HFpEF. Standard therapy for HF involved CV medications used in both groups of the EMPEROR-Preserved and DELIVER studies, including mineralocorticoid receptor antagonist (MRA), beta-blocker and renin-angiotensin system inhibitor (Anker et al., 2021; Solomon et al., 2022). Our model allowed us to simulate the costs, survival time and quality-adjusted life years (QALYs) across groups based on the simulated population.

### Transitional probabilities

The most pivotal input came from the EMPEROR-Preserved and DELIVER studies. For patients with HFpEF who received empagliflozin (10 mg daily) over a median of 26.2 months, 7.3% experienced CV death events, 8.6% had HHF events and 4.5% had urgent visits for HF. For patients with HFpEF in the comparator1 group, 8.2% experienced CV death events, 11.8% had HHF events and 7.2% had urgent HF visits (Anker et al., 2021; Packer et al., 2021). For patients with HFpEF who received dapagliflozin (10 mg daily) for a median of 2.3 years, 7.4% experienced CV death events, 10.5% had HHF events and 1.9% had urgent visits for HF. For patients with HFpEF in the comparator2 group, 8.3% experienced CV death events, 13.3% had HHF events and 2.5% had urgent HF visits (Solomon et al., 2022). Some other event rates not directly collected from the EMPEROR-Preserved and DELIVER research, such as CV death in hospitals and non-CV death, were derived from relevant published literature and national databases. The study by Goyal et al. reported the CV death rate in hospitals from 388,442,396 discharge records (Goyal et al., 2016). As no significant differences in non-CV diseases were found between the intervention and comparator groups in the EMPEROR-Preserved and DELIVER studies, the hazard ratio (HR) for non-CV death in the intervention groups compared with the comparator groups was established at 1.0, which was also associated with age dependence. Non-CV death of patients with HFpEF was collected from the 2020 China Mortality Surveillance Data Set (National Center for Chronic and Noncommunicable Disease Control and Prevention, 2019).The formula r = −1/t ln(1-S), P = 1-e^(-r*T) was used to calculate the transitional probabilities for each state of health (S represents the rate of the event, t represents the time and *p* represents the transitional probabilities) (Park et al., 2019) (Table 1).

**TABLE 1:** Selected model inputs.

Variables	Base	Range	Distribution	Source
Base probabilities(%)				
CV death	—	—	—	—
Comparator1 group	0.9749	0.8774–1.0720	Beta	Anker et al. (2021)
Empagliflozin group	0.8642	0.7778–0.9506	Beta	Anker et al. (2021)
Comparator2 group	0.9374	0.8436–1.0310	Beta	Solomon et al. (2022)
Dapagliflozin group	0.8322	0.7490–0.9154	Beta	Solomon et al. (2022)
Hospitalization for HF				
Comparator1 group	1.4275	1.2850–1.5700	Beta	Anker et al. (2021)
Empagliflozin group	1.0244	0.9220–1.1270	Beta	Anker et al. (2021)
Comparator2 group	1.5392	1.3850–1.6930	Beta	Solomon et al. (2022)
Dapagliflozin group	1.1985	1.0790–1.3180	Beta	Solomon et al. (2022)
Urgent visit for HF				
Comparator1 group	0.8520	0.7668–0.9372	Beta	Packer et al. (2021)
Empagliflozin group	0.5259	0.4733–0.5784	Beta	Packer et al. (2021)
Comparator2 group	0.2748	0.2473–0.3023	Beta	Solomon et al. (2022)
Dapagliflozin group	0.2083	0.1875–0.2291	Beta	Solomon et al. (2022)
CV death in hospital	13.175	7.5990–19.564	Beta	Goyal et al. (2016)
Non-CV mortality by age				
70–74 years	0.392	—	—	National Center for Chronic and Noncommunicable Disease Control and Prevention (2019)
75–79 years	0.624	—	—	National Center for Chronic and Noncommunicable Disease Control and Prevention (2019)
80–85 years	1.312	—	—	National Center for Chronic and Noncommunicable Disease Control and Prevention (2019)
85- years	1.626	—	—	National Center for Chronic and Noncommunicable Disease Control and Prevention (2019)
Utility				
HF with no events	0.817	0.717–1.000	Beta	Hong et al. (2018)
Hospitalization for HF	−0.1	−0.13–−0.08	Beta	Liang et al. (2018)
Cost				
Standard therapy	$125.49	$125.49–295.59	Gammma	Huang et al. (2017)
Empagliflozin	$ 56.70	$45.36–68.04	Gammma	Local data
Dapagliflozin	$ 58.31	$46.64–81.65	Gammma	Local data
Hospitalization for HF	$2662.60	$1404.98.73–3019.68	Gammma	Qiao et al. (2022)
Urgent visit for HF	$3918.75	$2669.91–4299.60	Gammma	Qiao et al. (2022)
Discounted rate	5%	0%–8%	Beta	Liu (2020)

CV, indicates cardiovascular; HF, heart failure.

### Cost

Given that the direct cost was calculated objectively and easily, we only included this cost. We needed to calculate the cost of standard therapy, HHF and urgent visits to HF. Standard therapy comprised conditional CV medications, including SAC/VAL, MRA, beta-blockers and angiotensin receptor blockers (ARB) or angiotensin-converting enzyme inhibitors(ACEI). Furthermore, Professor Huang calculated the cost of standard treatment collected from the database of national claims sampling (Huang et al., 2017). Given the last national negotiation price in 2023, the price of dapagliflozin was $0.6478 per 10 mg, the price of empagliflozin was $0.6300 per 10 mg and the price of SAC/VAL was $0.473 per 100 mg. Therefore, we collected a range of standard treatments for sensitivity analysis and costs of empagliflozin and dapagliflozin in each cycle. The cost of HHF and the cost of urgent visits for HF were derived from research on hospitalisation expenses for HF based on diagnosis-related groups, which was a disease classification method that comprehensively considered the diagnosis, severity and individual characteristics of the disease and had a significant effect in improving the comprehensive level of hospitals and controlling the unreasonable increase in hospitalisation expenses(Qiao et al., 2022). The costs in this study were presented in US dollars (the exchange rate of $1 US = 6.73 RMB) (The People’s Bank of China, 2022). In light of the healthcare Consumer Price Index based on the China Guidelines for Pharmacoeconomic Evaluations (2020), all costs were converted to 2022 values (Liu, 2020) (Table 1).

### Health-associated quality of life

Owing to limited research on the health utility of HFpEF in China, we used published studies in this investigation. Hong et al. used EuroQol-5 dimensions-5 levels to indirectly assess the health utility of all health statuses for HF in Korea, which demonstrated ideal validity, precision and dependability in patients (Hong et al., 2018). For HHF and the urgent visit for HF, we chose −0.1 as disutility based on the previous cost-effectiveness analysis for HF (Table 1) (Liang et al., 2018).

### Economic evaluation

The incremental cost-effectiveness ratio (ICER) was the main end point in our study. The ICER was obtained by adopting the difference in total cost divided by the difference in total quality-adjusted life years (QALYs) for the intervention and comparator groups. Secondary endpoints were total cost and QALYs, incremental cost and QALYs. Owing to the specified willingness to pay (WTP) to assess cost-effectiveness in China, the $12,032.10 per QALY associated with the one-time gross domestic product *per capita* of China in 2021 was chosen as the WTP threshold to determine whether the interventions (empagliflozin or dapagliflozin) had the cost-effective advantages (ICER less than or equal to the WTP threshold of $12,032.10 per QALY).

### Sensitivity analyses

Various sensitivity analyses were performed to evaluate the uncertainty of all model inputs. In one-way sensitivity analyses, key inputs varied within specified ranges, such as 95% confidence intervals (CIs), whereas other inputs remained constant. For transitional probabilities and costs without 95% CIs, a plausible range of ±10% and ±20%, respectively, was recommended. All ICERs were presented as a tornado diagram. In probability sensitivity analysis (PSA), we could simultaneously observe the impact of changes in all inputs on the ICER model. Random samples (1000 repetitions) of the key inputs with different distributions were used to calculate 1000 ICERs. The cost employed γ distribution, the utilities and transitional probabilities employed β distribution. The 1000 results were shown as scatter diagrams and cost-effectiveness–acceptability curves.

Several clinically related scenario analyses were performed to evaluate the impact of key structural assumptions. These enrolled different intervention prices (empagliflozin or dapagliflozin) (reduced by 20%, 40% and 60%), hospitalisation costs (reduced by 20%, 40% and 60%) and time horizon of the model (27 months, 5, 10, 15 and 20 years) along with the same CV death in both arms.

## Results

### Model validation

Our model results were compared with event rates from the EMPEROR-Preserved and DELIVER studies. At 27 months of follow-up, the intervention group (empagliflozin) had a CV death rate of 7.9% compared with that of 9.3% in the comparator1 group, the intervention group (dapagliflozin) had a CV death rate of 7.6% compared with 8.7% in the comparator2. The mean survival time of the intervention group (empagliflozin) and the comparator1 group was 15.25 years and 13 years, respectively. The mean survival time of the intervention group (dapagliflozin) and the comparator2 group was 15.75 and 14 years respectively. These data demonstrate that the results of our model were similar to those of the EMPEROR-Preserved and DELIVER studies.

### Base-case analysis

After simulating a 20-year lifetime horizon, a 72-year-old participant with HFpEF in the intervention group (empagliflozin) had a life expectancy of 7.32 QALYs compared with 6.88 QALYs in the comparator1 group and a 72-year-old HFpEF participant in the intervention group (empagliflozin) spent $8,250.55 compared with $6,626.97in the comparator1 group, with an ICER of $ 3,691.56 per QALY, which was lower than the WTP threshold of $12,032.10 per QALY. A 72-year-old patient with HFpEF in the intervention group (dapagliflozin) acquired a life expectancy of 7.43 QALYs compared with that of 7.09QALYs in the comparator2 group, and a 72-year-old patient with HFpEF in the intervention group (dapagliflozin) spent $8,153.14 compared with $6,151.00 in the comparator2 group, with an ICER of $ 5,907.79 per QALY, which was lower than the WTP threshold of $12,032.10 per QALY. These findings indicate that the interventions (empagliflozin or dapagliflozin) showed satisfactory cost-effectiveness (Table 2).

**TABLE 2:** The results from base-case analysis.

	Total cost($)	Total life years(QALY)	Incremental cost($)	Incremental life years(QALY)	ICER($ per QALY)
Intervention(empagliflozin) group	8,250.55	7.32	1,623.58	0.44	3,691.56
Comparator1 group	6,626.97	6.88	—	—	—
Intervention(dapagliflozin) group	8,153.14	7.43	2,002.13	0.34	5,907.79
Comparator2 group	6,151.00	7.09	—	—	—

### Sensitivity analysis

**FIGURE 2:**
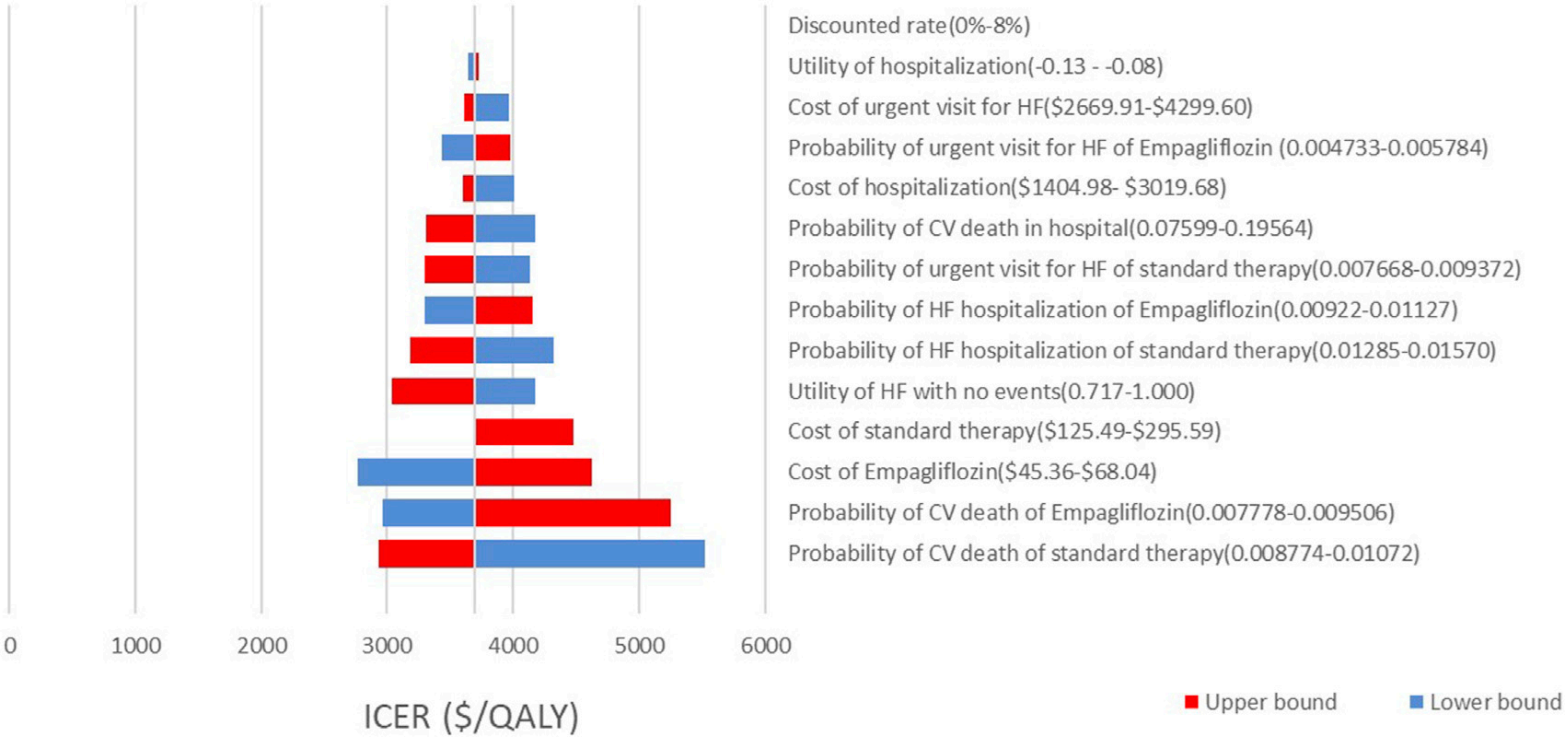
Tornado diagram showing one way sensitivity analyses of the intervention (empaliflozin) and the comparator.

**FIGURE 3:**
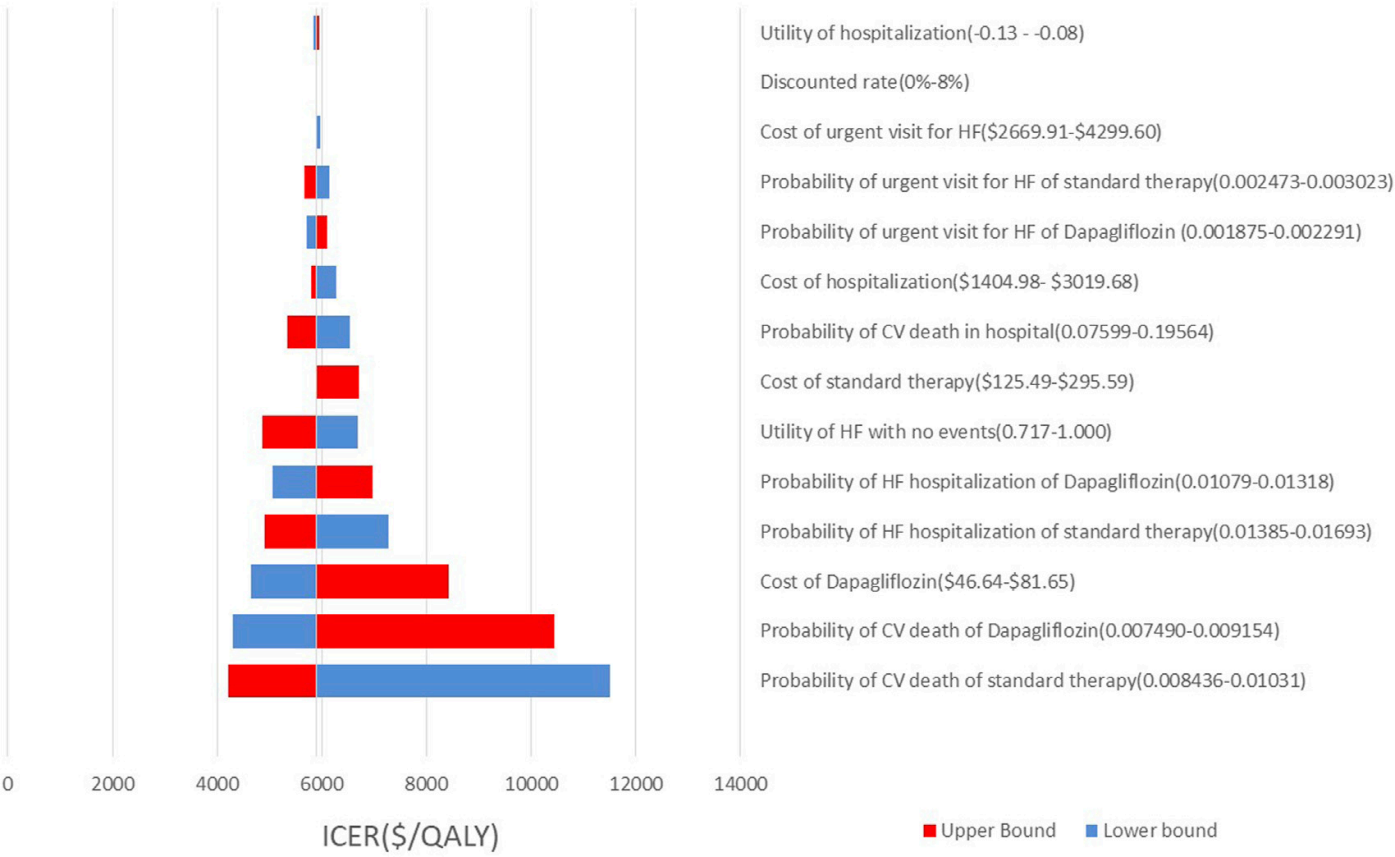
Tornado diagram showing one way sensitivity analyses of the intervention (dapaliflozin) and the comparator.

As shown by the results of the one-way sensitivity analysis (Figures 2, 3), the decision was most sensitive to CV death in the intervention and comparator groups, followed by the cost of empagliflozin and dapagliflozin, whereas other inputs had little effect on the decision. Additionally, when the transitional probabilities of CV death in the intervention and comparator groups changed from the lower limits to the upper limits, the corresponding ICER was still lower compared with the WTP threshold of $12,032.10 per QALY.

**FIGURE 4:**
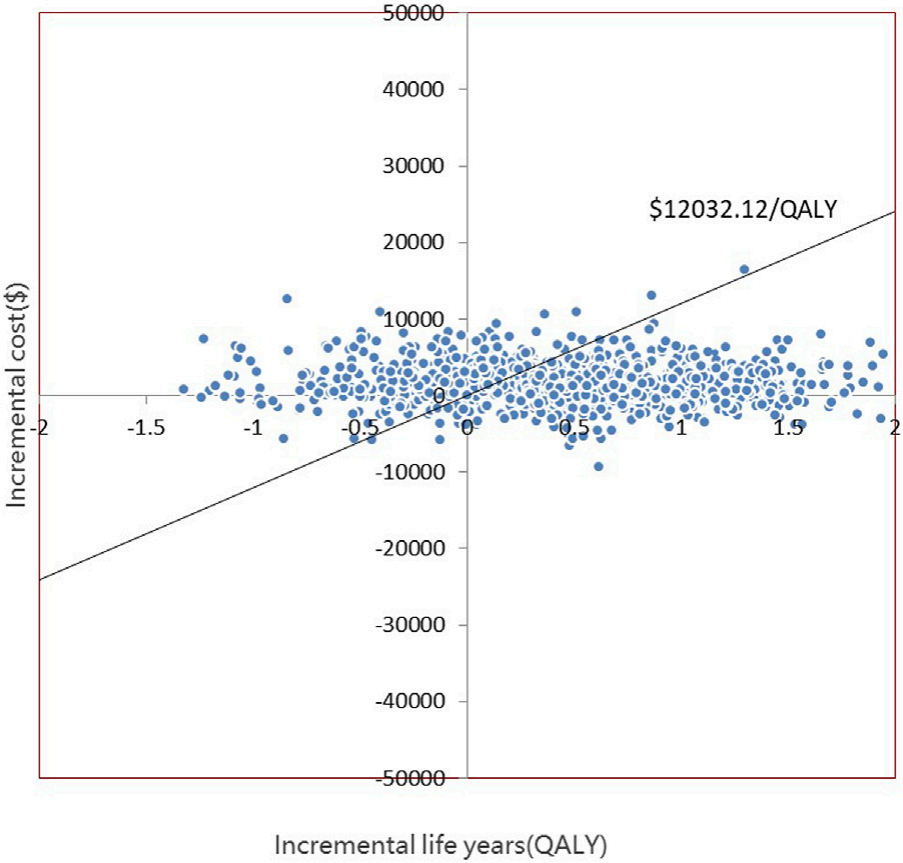
Scatter plot showing the incremental costs and incremental quality-adjusted life years of 1000 simulations for the intervention (empagliflozin) and the comparator.

**FIGURE 5:**
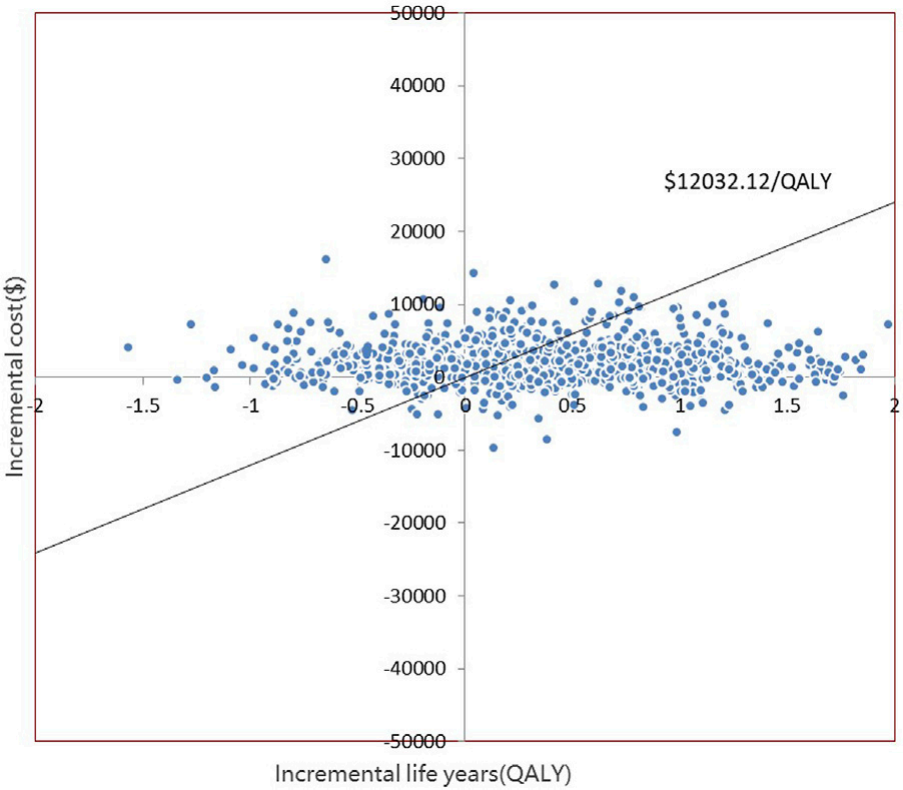
Scatter plot showing the incremental costs and incremental quality-adjusted life years of 1000 simulations for the intervention (dapagliflozin) and the comparator.

**FIGURE 6:**
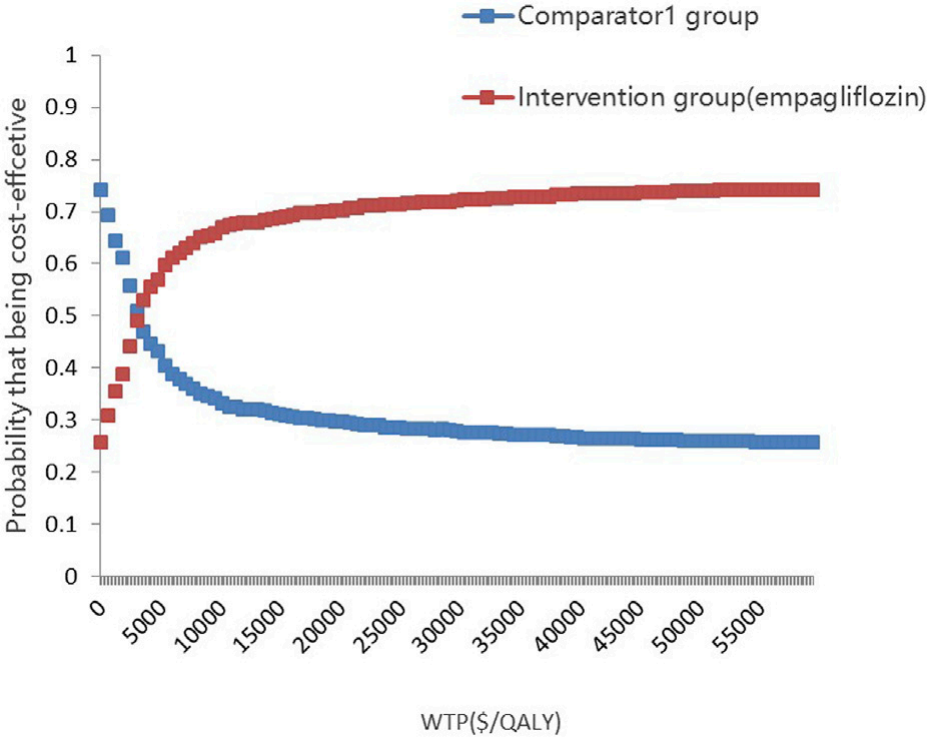
Cost-effectiveness-acceptability curves showing results from 1000 Monte Carlo simulations of the intervention (empaliflozin) and the comparator.

**FIGURE 7:**
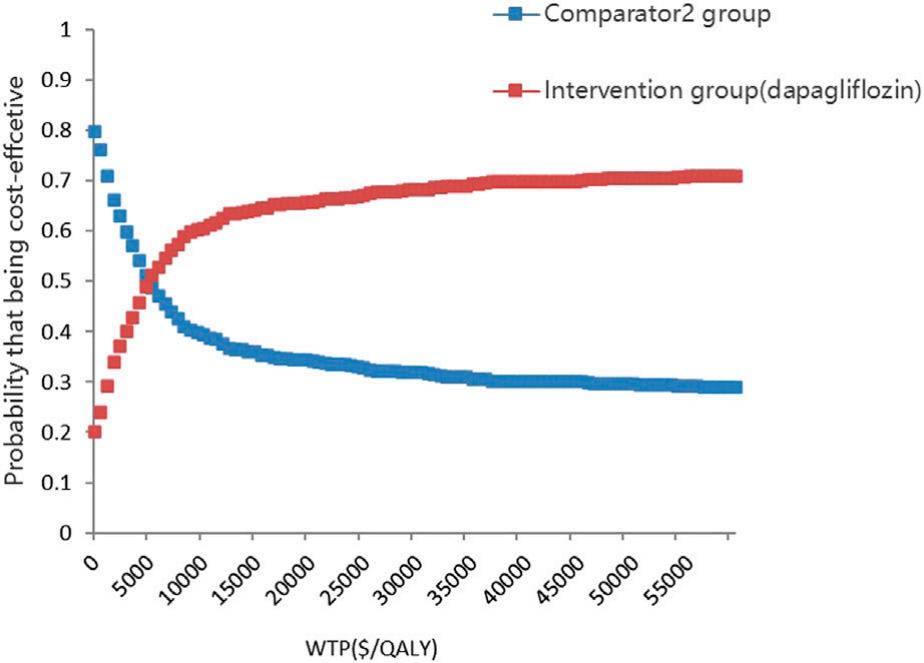
Cost-effectiveness-acceptability curves showing results from 1000 Monte Carlo simulations of the intervention (dapaliflozin) and the comparator.

The PSA is presented in Figures 4, 5. Most of the 1000 scatter points were found in the upper right quadrant, which demonstrates that the interventions (empagliflozin or dapagliflozin) generated an increased cost but acquired higher QALYs. Subsequently, when all key inputs were derived from their assigned distributions, the intervention group (empagliflozin) was cost-effective in 67.9% of 1000 Monte Carlo simulations, and the intervention group (dapagliflozin) was cost-effective in 62.2% of 1000 Monte Carlo simulations (Figures 6, 7).

The results are demonstrated in Table 3. We evaluated the effect of potential generic equivalents entering the market and found that their simulated prices significantly impacted the decision. The lower cost of the interventions (empagliflozin or dapagliflozin) brought greater pharmacoeconomic benefits, and the cost of hospitalization had a little impact the decision. However, the decision took into account the time horizon. If the time horizon was reduced from 20 to 5 years, the ICER of the intervention group (empagliflozin) and the comparator1 group increased from $3,691.56 per QALY to $7,718.52 per QALY gained, and the ICER of the intervention group (dapagliflozin) and the comparator2 group increased from $5,907.79 per QALY to $14,027.43 per QALY. Additionally, when the time horizon was 27 months, the ICERs were $13,067.49 per QAL Y and $25,083.47 per QALY, respectively. Similarly, when CV death in the intervention groups was the same as that in the comparator groups, the ICER was $6,083.37 per QALY and $13,454.68 per QALY, respectively.

**TABLE 3:** Scenario analyses.

Scenario	Empagliflozin	Dapagliflozin
	ICER ($ per QALY)	ICER ($ per QALY)
Base case	3,691.56	5,907.79
Price for empagliflozin or dapagliflozin	—	—
Reduced by 20%	2,761.08	4,647.86
Reduced by 40%	1,830.59	3,387.93
Reduced by 60%	900.10	2,128.00
Cost of hospitalization	—	—
Reduced by 20%	4,000.76	6,104.82
Reduced by 40%	4,309.95	6,301.85
Reduced by 60%	4,619.15	6,498.89
Time horizon	—	—
27 months	13,067.49	25,083.47
5 years	7,718.52	14,027.43
10 years	5,110.36	8,722.85
15 years	4,162.94	6,837.58
20 years	3,691.56	5,907.79
Same CV death	6,083.37	13,454.68

## Discussion

In the economic evaluation, we assessed the cost-effectiveness of the interventions (empagliflozin or dapagliflozin) for HFpEF in the Chinese healthcare setting. Interventions (empagliflozin or dapagliflozin) provided a high value in the treatment of patients with HFpEF, with $3,691.56 per QALY gained or $5,907.79 per QALY. A series of sensitivity analyses supported the certainty of the decision. Although the interventions (empagliflozin or dapagliflozin) were related to higher medical costs and had no additional benefits on CV death, this was offset by fewer HHFs and more QALYs compared with comparators in the base-case analysis (Chapman et al., 2004), which provided information for decision makers and healthcare payers.

CV death was the first sensitive factor for the decision in the current sensitivity analysis. It was expected that the EMPEROR-Preserved and DELIVER studies were not powered to evaluate the discrepancy in CV death. The EMPEROR-Preserved study found that empagliflozin had a similar effect on CV death among patients with HFpEF compared with standard therapy (HR, 0.91; 95% CI, 0.76–1.09) (Anker et al., 2021). The DELIVER research also showed that dapagliflozin was related to an insignificant decrease in CV death (HR, 0.88; 95% CI, 0.74–1.05) (Solomon et al., 2022). A meta-analysis that included EMPEROR-preserved and DELIVER studies also did not show a significant decrease in CV mortality (HR, 0.88; 95% CI,0.77–1.00) (Vaduganathan et al., 2022). When CV death in the intervention groups (empagliflozin or dapagliflozin) was the same as that in the comparator groups from the scenario analyses, the intervention (empagliflozin) was still cost-effective (ICER of $6,083.37 per QALY ≤ the WTP threshold of $12,032.10 per QALY). However, the intervention (dapagliflozin) was not cost-effective (ICER of $ 13,454.68 per QALY > the WTP threshold of $12,032.10 per QALY). Empagliflozin (HR, 0.71; 95% CI, 0.60–0.83) was more correlated with HHF reductions than dapagliflozin (HR, 0.77; 95% CI,0.67–0.89) in the treatment of HFpEF (Anker et al., 2021; Solomon et al., 2022), and empagliflozin reduced the appearance of urgent visits for HF (HR, 0.61; 95% CI,0.50–0.76) compared with dapagliflozin without reducing the urgent visits for HF (HR, 0.76; 95% CI,0.55–1.07) (Packer et al., 2021; Solomon et al., 2022). The researchers also explained why the intervention (empagliflozin) provided more pharmacoeconomic benefits than the intervention (dapagliflozin) without considering the CV benefits of empagliflozin and dapagliflozin. Further studies supporting the impact of empagliflozin and dapagliflozin on CV death among patients with HFpEF would provide greater economic value.

The cost of the intervention (empagliflozin or dapagliflozin) was the second sensitive factor for ICER. We explored the impacts of different prices of interventions (empagliflozin or dapagliflozin) on ICERs using scenario analyses by considering the introduction of generic drugs, which significantly affected the prices of empagliflozin and dapagliflozin. The emergence of new generic drugs that reduce medical costs would encourage patients with HFpEF to use SGLT2is. We found that the interventions (empagliflozin or dapagliflozin) were more cost-effective with a longer lifetime, that the interventions (empagliflozin) demonstrated optimal cost-effectiveness when the time horizon was 2.5 years and that the interventions (dapagliflozin) demonstrated optimal cost-effectiveness when the time horizon was 6.25 years. These findings placed significant emphasis on the fact that patients with HFpEF prolonged their use of SGLT2is owning to an increase in comorbidity for a significant number of patients.

Owing to the extensive use of SGLT2is in HFrEF populations, the EMPEROR-Reduced and DAPA-HF trials provided clinical evidence that SGLT2is could reduce CV mortality or HHF (Mcmurray et al., 2019; Packer et al., 2020). Several cost-effectiveness analyses of SGLT2is in HFrEF have been conducted in middle-income countries as well as high-income countries, such as China, Thailand, the Philippines, the United States and Egypt (Yao et al., 2020; Krittayaphong and Permsuwan, 2021; Mendoza et al., 2021; Parizo et al., 2021; Savira et al., 2021; Abdelhamid et al., 2022).In these studies, SGLT2is showed the cost-effective advantage in HFrEF among these countries. Given the clinical efficacy of SGLT2is in HFrEF populations, the additional cost of SGLT2is could be ignored. However, the results of the cost-effectiveness analyses of SGLT2is in HFpEF were not satisfactory. The combined use of empagliflozin with standard therapy in HFpEF brought pharmacoeconomic benefits in China and Australia (Zhou et al., 2022; Lou et al., 2023), but provided low value in the United States and Thailand (Krittayaphong and Permsuwan, 2022; Zheng et al., 2022), which was closely associated with the healthcare system, national economic status and WTPs. Cohen et al. also explored the pharmacoeconomic benefits of empagliflozin and dapagliflozin in HFpEF in the United States, indicating that adding SGLT2is to standard care was of intermediate or low economic value (Cohen et al., 2023). Differing from our study, their study chose the relevant data from the meta-analysis of the EMPEROR-Preserved and DELIVER studies instead of individual clinical trials, ignoring the unique efficacy of empagliflozin or dapagliflozin. To the best of our knowledge, there were no pharmacoeconomic studies on cost-effectiveness analyses of dapagliflozin in HFpEF in China.

This discrepancy might demonstrate significant differences between the HFrEF and HFpEF populations. Among older individuals, those with HFpEF tended to have more comorbidities, more deaths from non-CV causes and lower CV deaths than those with HFrEF (Chan and Lam, 2013). Thus, the pharmacoeconomic benefits of SGLT2is for those with HFpEF only included the reduction of HHF and urgent visits for HF without assuming any CV mortality benefits of this intervention. This finding explains why SGLT2is was more cost-effective for HFrEF than HFpEF. HFpEF is a crucial public health issue that affects millions of people worldwide. The financial burden imposed by this condition was mainly derived from the high incidence and prolonged hospitalisation for high comorbidities. Furthermore, 67.4% of patients with HFpEF in China had the three most common comorbidities, including hypertension, atrial fibrillation and coronary heart disease, which were also the main risk factors for hospitalisation for HF (Cai et al., 2022). Owing to interventions (empagliflozin or dapagliflozin) that demonstrate important benefits in terms of clinical outcomes, the use of SGLT2is could be a novel therapeutic strategy for HFpEF.

However, the following limitations should be considered when interpreting the results of our research. First, considering the data from the EMPEROR-Preserved and DELIVER studies over a shorter period, we assumed that the efficacy and QALYs of SGLT2is had lasted 20 years. Our decision should be updated when follow-up data are available. Second, as each input in the model contained an uncertainty, we performed a series of sensitivity tests. Our decision remained unchanged although each input varied across a reasonable range or assigned distributions. Third, although the data collected by us were derived from an authoritative database, the built model could not reflect real practise, such as patient adherence and tolerance to SGLT2is. Fourth, we did not enrol any analyses of the renal protective effect of SGLT2is because the renal endpoints were small and non-significant, but could improve long-term results independent of the benefits observed in the EMPEROR-preserved and DELIVER studies. Finally, the decisions of this cost-effectiveness analysis were suitable for the Chinese setting; other countries with different populations, medical costs and healthcare systems should be cautious in their execution.

## Conclusion

In the Chinese healthcare system, the interventions (empagliflozin or dapagliflozin) for HFpEF were cost-effective compared with comparators, as proven by the quantitative evaluation of lifetime benefits and costs using our model. Our findings possibly provide new insights for decision makers and healthcare payers, with a focus on the cost-effectiveness of SGLT2is; however, more studies based on real-world data are required.

## Data Availability

The original contributions presented in the study are included in the article/Supplementary material, further inquiries can be directed to the corresponding author.
